# Report of the Committee on the Scottish Health Services

**Published:** 1936

**Authors:** 


### REPORT OF THE COMMITTEE ON THE SCOTTISH HEALTH SERVICES.

BY

THE EDITOR.

---

In our Spring issue this year (Vol. LIII, 199, p. 39) we were privileged to publish a Synopsis of the Memorandum submitted by the Voluntary Hospitals in Glasgow and the West of Scotland to the Departmental Committee of Enquiry into the Scottish Health Services. The Report of the Committee has now been published,* and it is probable that legislation will follow which may foreshadow similar legislation for England and Wales.

Part III of the Report deals with "Medical and Allied Services." On the "Rôle of the Family Doctor" the Committee finds that the work of the general medical practitioner is not effectively linked up with that of the main statutory services, and agrees that this link is vital to any comprehensive policy for the promotion of the health of the people. The family doctor should be in ordinary practice the health adviser, and the training of the medical student should be such as to produce the preventive outlook. Without the assistance of the family doctor as health adviser the statutory medical services cannot, to the full extent, promote and safeguard the health of the people.

* *Report of the Committee on Scottish Health Services.* Department of Health for Scotland. Price 6s.

The Committee has no doubt that large numbers of the population who are not included in the National Health Insurance Scheme do not receive adequate medical attention. One effect of this is to increase the pressure on the hospital out-patient departments, another is to add to the cost and reduce the efficiency of the statutory medical services.

In spite of school medical services many children are not treated at all, sometimes because of parental neglect, but more generally because of the cost of obtaining appropriate medical treatment. "We find," says the Report, "that inadequacy of general medical attendance adds to the cost, interferes with the efficiency, and prevents the sound development of many of the medical services provided by statute." It is imperative for the State to frame a policy to meet the needs of the dependants of insured persons and others to lay down the lines along which the medical services should develop.

While the plan of whole-time medical officers of health and certain other specialists working for the local authority is a sound and valuable principle the Committee regards it as obvious that the development of whole-time service into the whole field of medical practice would be unsatisfactory. The co-ordinated and extended general practitioner service should be built up on the basis of contract with the private general practitioner, and remuneration should be by capitation fees.

The maternity service should be based on the doctor and midwife acting in concert, supplemented by consultants and institutional facilities. But more practical training of doctors and midwives, along with other measures, are needed to raise the general standard of midwifery practice.

Child welfare work is seriously handicapped by the inadequacy of arrangements for home medical and nursing attention. The general practitioner should become an integral part of child welfare service and schemes should be developed in co-operation with him. So, too, the work of the school health service is seriously hampered by the inadequacy of medical supervision at home: here again domiciliary attention and supervision must be secured by co-operation between local authorities and family doctors. The Poor Law Medical Service is found unsatisfactory, and in the view of the Committee, cannot be improved so long as a separate domiciliary medical service is maintained for persons only when they are destitute. "Poverty," says the Report (with Scottish forthrightness), "must not be permitted to interrupt the continuity of medical supervision by the family doctor." The Committee, hoping for a review of the law *of settlement and recourse* would welcome any changes that will make treatment available to all alike without any distinction between the general public and the poor.

As regards the National Health Insurance Scheme the Committee is convinced of its success. The proposals contained in the Report contemplate a co-ordinated and complete medical service in which the general practitioner, acting normally as family doctor, will act in liaison between the homes of the people and the facilities provided by statutory and other agencies.

The hospital services are the subject of recommendations, some of which involve drastic alterations. There has been for a long time a serious shortage of hospital facilities which militates against the ideal of early treatment, and causes much loss of working time and avoidable expenditure from insurance and other funds. An adequate hospital service is essential for the public health. The State should foster the voluntary hospital system, but it would be unwise to endeavour to induce the voluntary hospitals to extend their financial commitments much further than at present.

The hospital services should be developed by co-operation of voluntary and statutory hospitals and the public authorities, but, in the main, financial responsibility for making up the shortage should be placed on the local authorities, who should be assisted by special Treasury grants for this purpose.

The State should recognize the services of the teaching hospitals in respect of their providing teaching facilities, and for this purpose a "teaching facilities grant" should be made available. Voluntary hospitals should be exempted from payment of legacy and succession duties, and should be granted remission from rates.

To secure an adequate hospital service in modern conditions and to safeguard voluntary hospitals the hospitals of all kinds must be viewed as a whole, and over wide regions, and must be regarded as one service. For the development of hospital services on co-operative lines and in the interests of the voluntary hospitals, it is essential that the voluntary hospitals should be recognized as an integral part of the national health services, and they should, as a corollary, accept a measure of supervision and guidance from the Department of Health.

The Report is too long to be discussed in detail. The foregoing abstract presents its recommendations as to General Practitioner and Hospital Services. Part III also deals with School Health Service, Infectious Diseases, Tuberculosis, Venereal Diseases,

Mental Health Service, Industrial Health Services, Nursing and Allied Services, Dentistry, Clinics and Diagnostic Centres, and Pharmacy. Part I considers Social and Economic conditions in relation to the Public Health. Part II deals with Heredity, Food, Education, and what are called "Environmental Services" such as Housing, Water Supply, Drains and Sewers, Cleansing and Scavenging.

The comprehensive nature of the Report is thus evident, and its conclusions are supported with considerable unanimity. There is no minority report, and the "reservations" made by individual members of the Committee only serve to emphasize the approval which the Report, as a whole, commands. Its wise recommendations seem to require very little alteration to render them applicable to England as well as Scotland.

